# Publish or perish: a provocation

**DOI:** 10.1590/S1516-31802008000300013

**Published:** 2008-05-01

**Authors:** Eduardo Katchburian

## To be or not to be cited, that is the question modified from Shakespeare

I would most probably be lying if I said that I do not care whether my research papers are cited or not by my scientist colleagues. I do care. We all care. We care because like all human beings we want recognition: in short, we all want to be loved by everybody all the time. Human beings, unlike other species, are extremely insecure and unless they are told again and again that they are wonderful, they get depressed and become suicidal. This reminds me of a movie by the Marx brothers (not Karl) in which someone tells Groucho that he is the most wonderful chap and he has never met anybody so marvelous and so wonderful! Groucho looks at us and says, “Well, I could talk and listen to this chap for the rest of my life”.

Nowadays, brainwashed as we are by the Americans, we measure how much we are loved by counting how many times we appear on the list of references of a paper. Indeed the whole world, except Al-Qaeda and Bin Laden, has been steamrolled into believing that our scientific worth can be measured by the number of citations we have and by the impact of the journal we publish in. And the immediate effect of such obsession is that we no longer think or do proper science. We publish! Everything is geared to publications. We look for research themes that are likely to yield publishable results or from which the results are predictable and thus no longer constitute research. And we try to do research that will result in a certain number of papers per year. Long-term projects are not on. We can no longer afford to take risks because whatever we do must be publishable. Research is becoming a factory assembly line. Thus, we end up with a massive pile of rubbish, of papers, mere potboilers that will burn on top of a rubbish tip and evaporate into oblivion.

In the beginning, it was the number of papers that counted. Soon, people learned how to get their names on papers. People learned all sorts of tricks to get their names on papers they knew very little about. For example, colleagues exchanged courtesies: you put my name in your paper and I will do the same, and we will double our number of publications. Also, instead of publishing complete stories, i.e. all results, people divided them up into three or four papers to boost the total number. A wonderful way of circumventing the system. The result is that there are people with hundreds of papers! And they are proud to have hundreds of papers published, or in fact to have their names among the authors, even if their contribution was negligible or none at all. I am sure that if we were to squeeze the whole lot there would only be a few drops of useful information left. Long-term projects are not on, let alone originality, creativity or serendipity.

In this day and age of multidisciplinary work, there are papers with an enormous number of authors and it is impossible to know who has contributed with what. Are authors only those who appear first and last? What about those in the middle? Indeed, there are no hard and fast rules about the order in which authors appear on a paper. People who are more radical say that you are an author only if you are capable of delivering a full and complete lecture or talk on the whole paper. I cannot see what satisfaction people may derive from having their name stuck on a paper they know little or nothing about. And how do you compare someone who has published only 10 or so papers but is the first or last author in all of them with someone who published 500 papers but never appeared as a first or last author? In an attempt to be fair, some departments adopted a rotation system in which everyone involved in the project would, sooner or later, appear as the first author; others considered listing authors in alphabetical order. The question of authorship remains controversial and, indeed, painfully intractable. And let us not forget that it is much easier to get a paper published if the editor of the journal happens to be a friend!

Fortunately, it was soon realized that numbers of publications alone were not sufficient to evaluate a scientist’s worth. It was necessary to find a better way, and along came the citation index, i.e. counting the number of times we are cited. The citation index is obtained from a database produced by the Institute of Scientific Information (ISI) in the United States. The references are organized in the database to show how many times each paper has been cited within a certain period, and by whom. It is based entirely on the list of references compiled by the authors and as such it is strongly biased and, I dare say, almost arbitrary. And all publications not listed on the ISI database simply do not exist. Anything published in any language other than English, i.e. non-English, is thrown into the dustbin of science.

There is no hard and fast rule about the papers we choose to cite. We cite or do not cite virtually as we wish. Gone are the days when we tried hard to give the correct credit to people. I have been cited, more often than not, for something I have not done or for the wrong reasons. People vary a lot in the way they prepare their list of references. I must confess that I always try to cite friends and people I know, and of course my own publications. It is human nature! My citation index has always improved whenever I have had the chance to have a cup of coffee or a glass of wine with colleagues at scientific meetings. Some authors compile a long list; others only cite papers from the last five years. Also, some journals discourage long lists of references. Review articles are often cited and as such may conceal original papers. Some methodological papers are often cited for a very long time as a matter of habit. So, I think I would not be too wrong in saying that there is a strong element of subjectivity. We obtain a citation index, which is based on a list of papers selected, I dare say, almost arbitrarily. We obtain a numerical value, supposedly objective, based on a non-objective selection method. After all, for example, is it better to be cited once in a prestigious journal or many times in a second-rate journal? And we may be cited because we are wrong! In addition and more important is the fact that our colleagues from the Anglo-Saxon world in particular have a strong bias against research done in third world countries and therefore tend not cite them. There is a credibility gap between developed and underdeveloped countries. I can therefore say that papers originating in the third world are, most probably, undercited.

In view of the difficulties above, a new way of assessing scientists came about: the so-called impact factor, i.e. how often the journal you publish in is actually cited by others. The journal is therefore as good as its impact factor. But the impact factor is also based on the original list of references compiled by the authors, i.e. we cite the journals we decide to cite. Some arrogant and conceited scientists only cite articles published in top journals. Uncited articles may be given full credit for the impact of few highly cited papers. The correlation between journal impact and actual citation index is not very good. The academic community and administrators have equated impact factor as a measure of excellence without understanding how it is arrived at. I may, for example, have a paper published in a high-impact journal and never be cited by anybody. I could go on forever pointing out the pitfalls of the impact factor.

More recently, a new attempt has been made to overcome the difficulties of the citation index and impact factor. A new calculation, the so-called “h-index” has been created. I am afraid I have not been able to fully grasp how it is calculated, but again, it is biased because it is based on the list of references compiled by the authors. The “h-index” is claimed to be better than any other numerical system. However, I do not believe we will find a formula or a coefficient that will take into account all the variables and complexities involved in scientific research.

The history of science is full of examples of scientific findings that have been ignored for a long period of time. It is notoriously difficult for contemporaries to judge each other and decide what is important or not. What unsuspected developments may come out of Pandora’s box of science!

The best and classic example of failure to recognize a fundamental discovery on time is that of the Austrian monk Gregor Mendel. It was only nearly 40 years afterwards that his work and findings on peas were recognized by deVries and Correns as containing the fundamental laws of inheritance, i.e. genetics! It took Mendel eight years to do his work and apparently, he published only two papers. There was total failure among his contemporaries to understand and grasp the relevance of Mendel’s work. Would anyone give money to a reclusive monk to work on peas? When Mendel died, in 1884, he was a scientific nonentity. Impact factor zero!

Another classic example is the discovery of penicillin by Alexander Fleming (Nobel prize, 1945). The discovery of penicillin was not the result of a carefully prepared scientific project. It was an accident. A bacterial plate was accidentally contaminated with mould and Fleming observed that, all around the mould, the bacteria had been killed. Staphylococci were undergoing lysis around the contaminating colony. Most of us would have thrown the plate away. And, no doubt, Fleming was helped by the damp and dreary English weather, ideal for growing mould. Nobody paid much attention to his papers published in 1928, and it was nearly 12 years later that his papers were rediscovered by Florey and Chain (Nobel prize, 1945). The discovery of penicillin proves the point that it is when experiments go wrong that we find things out! Impact factor zero!

Another, more recent example of failure of contemporaries to appreciate a non-intuitive discovery was that of *Helicobacter pylori* as the causative agent of gastric (peptic) ulcers. Marshal, a young Australian doctor with no experience in research, noticed that patients with peptic ulcers undergoing a course of antibiotic treatment for some unrelated infectious disease improved considerably or were cured of the ulcer. Marshal and the pathologist Warren (Nobel prize, 2005) found the *Helicobacter bacterium* in the lesions, and proposed that bacteria were causing the ulcer. Nobody believed them. It was unthinkable and against the mainstream of ideas about ulcers, which were regarded as due to excessive secretion of acid, which in turn was related to stress and other fanciful psychological factors. The prevailing idea was that no bacteria could survive the strongly acid environment of the stomach. To persuade the scientific community that he was right, Marshal decided to do a self-experiment. He swallowed a cocktail containing large numbers of *Helicobacter* and got very sick and developed symptoms of dyspepsia. A biopsy showed lesions containing bacilli. After antibiotic treatment he was cured! Impact factor zero!

Even the seminal paper by Watson and Crick, published in Nature in 1953, on the DNA double helix, was received with indifference in some quarters and regarded as a gross oversimplification. It was only nine years later, in 1962, that they were awarded the Nobel Prize.

Sidney Brenner (Nobel prize, 2002) said, quite rightly: “What matters absolutely is the scientific content of a paper, and nothing will substitute for either knowing or reading it”. Scientific quality can only be measured by qualified experts reading the full paper, wherever published.

In fact, it is not possible to predict radical scientific innovations or fundamental conceptual changes. We may be able to predict inventions based on current scientific knowledge but we have no idea what the future will bring. That is the nature of science.

